# Coexpression analysis of large cancer datasets provides insight into the cellular phenotypes of the tumour microenvironment

**DOI:** 10.1186/1471-2164-14-469

**Published:** 2013-07-11

**Authors:** Tamasin N Doig, David A Hume, Thanasis Theocharidis, John R Goodlad, Christopher D Gregory, Tom C Freeman

**Affiliations:** 1 Centre for Inflammation Research, University of Edinburgh, The Queen’s Medical Research Institute, 47 Little France Crescent, Edinburgh EH16 4JT, UK; 2 Department of Pathology, Lothian University NHS Trust, Western General Hospital, Crewe Road, Edinburgh EH4 2XU, UK; 3 The Roslin Institute, R(D)SVS, 741 University of Edinburgh, Easter Bush, Midlothian, Scotland EH25 9RG, UK

**Keywords:** Cancer, Transcriptomics, Coexpression, Disease networks, Clustering, Modules, Gene signatures, Stroma

## Abstract

**Background:**

Biopsies taken from individual tumours exhibit extensive differences in their cellular composition due to the inherent heterogeneity of cancers and vagaries of sample collection. As a result genes expressed in specific cell types, or associated with certain biological processes are detected at widely variable levels across samples in transcriptomic analyses. This heterogeneity also means that the level of expression of genes expressed specifically in a given cell type or process, will vary in line with the number of those cells within samples or activity of the pathway, and will therefore be correlated in their expression.

**Results:**

Using a novel 3D network-based approach we have analysed six large human cancer microarray datasets derived from more than 1,000 individuals. Based upon this analysis, and without needing to isolate the individual cells, we have defined a broad spectrum of cell-type and pathway-specific gene signatures present in cancer expression data which were also found to be largely conserved in a number of independent datasets.

**Conclusions:**

The conserved signature of the tumour-associated macrophage is shown to be largely-independent of tumour cell type. All stromal cell signatures have some degree of correlation with each other, since they must all be inversely correlated with the tumour component. However, viewed in the context of established tumours, the interactions between stromal components appear to be multifactorial given the level of one component e.g. vasculature, does not correlate tightly with another, such as the macrophage.

## Background

In recent years the field of cancer research has seen an increasing number of large gene expression studies of primary human tumours. Analysis of these datasets has tended to focus on the identification of markers able to divide disease samples into prognostically relevant classifications [1-5]. In the seminal paper by Alizadeh *et al.*[6] they were able to subdivide lymphomas on the basis of their gene expression profiles and thereby associate specific genes with the tumour’s clinical characteristics. Subsequently, numerous studies have attempted to classify other tumour types based on their gene expression profiles and others to stratify patients into the most appropriate treatment group. The latter whilst not yet driving therapeutic options, clearly has potential implications for individualised patient therapy [4,7]. These studies have generally focused on the identification of groups of differentially expressed genes that can be used to divide tumours into subgroups using statistical approaches. Such predictive gene signatures are frequently composed of genes with no obvious shared biological function. Indeed, there may be a number of signatures derived for essentially the same purpose that share few if any genes in common [8].

An alternative approach is to generate signatures that reflect a specific biological process or outcome [9-11] or sets of coexpressed genes based upon correlation matrices [12-14]. One issue complicating analysis of any cancer gene expression data is the heterogeneity of samples. The tumour cells themselves differ not only in the nature of the mutation(s) that have driven them, but also the genotype of the patient and the treatment that they have received. A significant proportion of a tumour mass is comprised of stromal cells [15]; these non-transformed cells forming the microenvironment in which tumour cell growth is contained and supported. Indeed, the tumour stroma is increasingly seen as an alternative target for therapeutics with potential treatments targeting angiogenesis [16,17], the extracellular matrix [18] or immune cells [16,19].

**Figure 1:**
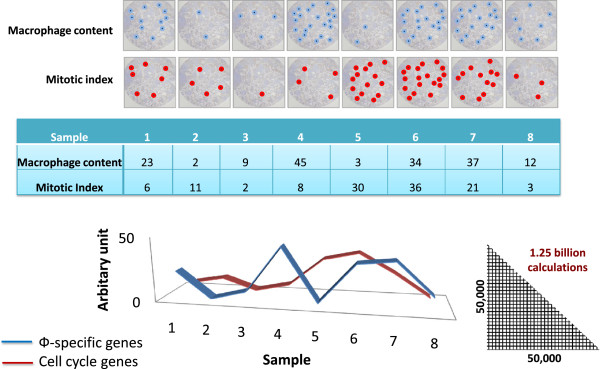
**Rationale behind the study. The relative number of a specific cell type or activity of certain pathways will vary across a collection of individual tumours.** For example, the macrophage content (Φ) will differ in every tumour and so therefore will the mRNA level of macrophage specific genes (in blue). Similarly in every tumour at the point it is sampled the number of cells in mitosis (the mitotic index) will differ and this will be reflected in different levels of expression of cell cycle genes (in red). As a result the expression level of genes specifically expressed by those cells or associated specifically with the pathways will vary accordingly. By calculating the correlation coefficient between every gene on the array and every other gene on the array it is possible to calculate a correlation matrix that includes all these correlation coefficients. Graphs are then used to visualise relationships above a given correlation threshold and clustering used identifying groups of co-expressed genes.

One approach to analysis of the cancer versus the stromal components in a tumour is to employ laser capture microdissection e.g. [20]. Here we present an *in silico* approach to dissecting the expression profiles of individual cell types in the tumour stroma, as well as the cancer cell component. We have developed a computational framework and associated tool that now supports visualization and clustering of very large correlation networks derived from microarray expression data [21,22]. The approach takes advantage of the heterogeneity of tumour samples. The underlying premise of this approach is that the expression of genes specifically associated with a given cell type or pathway will increase or decrease with the relative abundance or activity of those cells/pathways within a given sample, either because of genuine biological variation or random sampling of different regions of the tumours (Figure 1). The relative significance of correlation increases with the size of the dataset, as the probability of coexpression occurring by chance decreases. Modelling these associations as a graph brings together groups of functionally associated genes which share similar expression profiles such that they form cliques of high connectivity in a graph. We have recently confirmed this hypothesis through the meta-analysis of large collections of expression data derived from many different populations of mouse cells [23,24], pig tissues [25] and clinically derived samples [26]. Here we demonstrate using individual cancer datasets that global expression patterns can be divided into biologically meaningful clusters defining tumour cell and stromal elements, and also that many of these gene signatures are conserved across multiple unrelated human cancer datasets.

## Results and discussion

### Network topology

**Figure 2:**
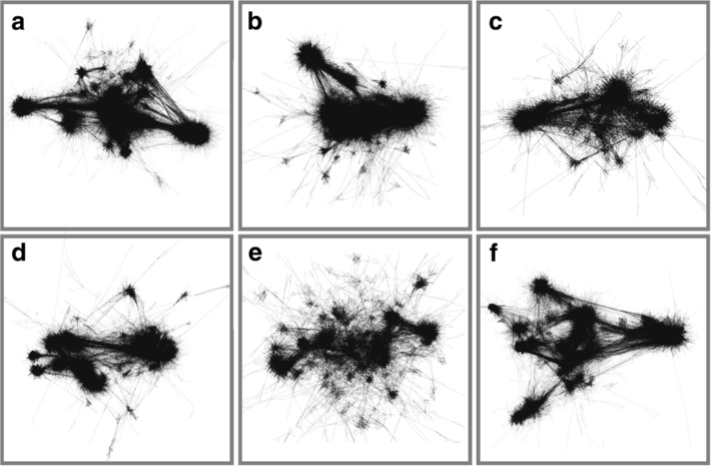
**Network graphs derived from six cancer datasets; a) breast carcinoma, b) colorectal carcinoma, c) DLBCL, d) glioma, e) ovarian carcinoma and f) testicular germ cell tumours.** Each dataset studied had its own idiosyncrasies owing to the tumour-specific biology each represents and the high degree of inherent variation in gene expression data derived from cancer samples. In order to visualise and analyse a large proportion of the expressed genes in these samples we aimed to construct graphs of data derived from individual cancer types based on just under half the transcripts represented on the chip (18,000-23,000 probesets). For these reasons relatively low correlation thresholds were used for graph construction i.e. between r = 0.65-0.75. The resultant graphs of individual cancer datasets are highly structured and composed of a large number of nodes and edges (18,934 - 23,015 nodes, connected by between 268,471 - 954,082 edges, see Table 1 for details).

Following download of all cancer data it was subjected to rigorous QC as poor quality data can have a significant and detrimental effect on correlation network topology. Only data that passed QC was used to construct the large networks described here. Network topology was visibly complex (Figure 2) and unsupervised cluster analysis using the Markov clustering (MCL) algorithm[27] defined cliques of highly connected nodes in all graphs. Each clique (cluster) represented transcripts whose expression patterns were highly correlated across the dataset. These were surrounded and linked by sparser network structures. GO and pathway enrichment analysis was able to demonstrate functional enrichment in many of the clusters, but was generally poor in identifying clusters associated with the specific cell types from which some of these signatures were clearly derived. We therefore supplemented this analysis by comparison with gene sets (clusters) derived from datasets of isolated tissues and purified cell populations [28,29] and mining of the literature. All graphs described in this work are available from the website http://www.OncoGraph.org which supports the direct visualization of graphs in BioLayout *Express*^3D^ using Java web start technology.

### Technical replicates and functionally related genes are closely associated in the graph network

**Figure 3:**
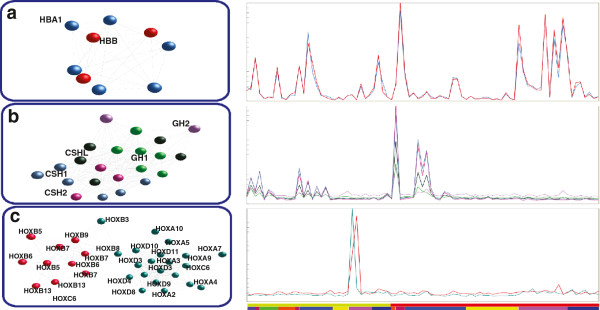
**Clusters of transcripts (left) derived from the testicular cancer dataset and (right) associated expression profiles (average signal per gene or cluster).** The individual tumours (represented along the y-axis) were grouped by mixed (green in upper bar) or pure histological subtype (red in upper bar) and then by components present (coloured blocks in lower bar) **a)** Haemoglobin cluster containing data derived from 5 probesets designed to HBA1 and 3 designed to HBB. The haemoglobin locus cluster is found to be present in many human expression datasets and is often unconnected to any other genes. **b)** Cluster of somatotrophin genes whose expression is normally tissue specific and limited to pituitary and placenta, shown here to be expressed predominately in tumours containing elements of choriocarcinoma, a tumour formed of malignant trophoblast cells, the normal equivalent of which are involved in placenta formation. **c)** HOX genes found to be expressed only in two teratomas with secondary carcinomatous transformation. Interestingly one of these tumours shows a high degree of up regulation of one group of primarily HOXB genes and the other a mix of HOXA/C/D genes.

Data derived from different probesets designed to the same gene, genes in the same loci and functionally related genes were frequently found to be connected within the graphs. For example in the testicular dataset the six probesets designed to haemoglobin alpha (*HBA1*) and three designed to haemoglobin beta (*HBB*) clustered together reflecting the known co-regulation of these loci (Figure 3a). Likewise the multiple probesets for the growth hormone genes, *GH1* and *GH2*, were closely associated within the testicular network graph alongside the chorionic somatomammotropin hormones (*CSH1*, *CSH2*, *CSHL1*) which are all sited at the same loci on chromosome 17 (Figure 3b). Furthermore, two clusters of Hox genes were found to be co-expressed in certain testicular cancers (Figure 3c). Finally, markers of monocyte/macrophage populations *CD14, CSF1R* and *CD163* were all expressed at highly variable levels across individual tumours but exhibited very similar profiles across the dataset. Indeed, these markers were always closely associated within all graphs and were usually all present in a single MCL cluster. Furthermore, these clusters were also enriched with many other genes also known to be expressed in macrophages (for example gene list of a macrophage cluster derived from DLBCL, see Additional file 1). In a cluster such as this in which many of the genes can be associated with a given cell type, the cluster provides a unique insight into the functional profile of the cell within the tumour. Although many of the genes in such clusters are not recognised markers of these cells and are therefore only characterised as such by the principle of ‘guilt by association’, they may be of significant interest in terms of defining the functional activity of cells or as potential targets for manipulating cell function.

### Individual tumour datasets form unique network structures related to the specific mix of cell populations

**Figure 4:**
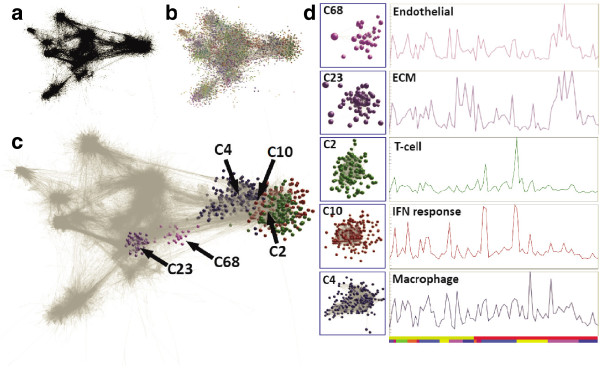
**Network derived from testicular cancer dataset (Pearson correlation threshold r = 0.75). a)** Network with only edges showing allowing visualization of the inherent complex topology of the graph and **b)** with nodes shown where nodes are coloured according to cluster membership. **c)** Graph with selected clusters shown and **d)** the average expression profile of genes within those clusters. (Cluster colour code is maintained across graphs in this figure). Cluster 68 is highly enriched with endothelial marker genes and cluster 23 contains many transcripts known to be associated with extracellular matrix. The last three selected clusters can be associated with different aspects of the immune infiltrate in these tumours. Cluster 2 contains many know markers of T-cell and B-cells, cluster 4 (also shown in Figure 2) is enriched with many known macrophage expressed genes and cluster 10 is highly enriched with many interferon response genes.

In previous studies of mouse primary cells and tissues [21,23,24] we were able to identify clusters associated with specific cell populations and others that reflected particular cell functions. This was possible because cells vary in their relative activity of different aspects of cell biology e.g. growth and proliferation, metabolism, protein synthesis and secretion, endocytosis, motility etc. Similarly, in this analysis many clusters showed clear functional enrichment of genes encoding proteins associated with a cell-specific pathway or cellular process (Figure 4). Some clusters were common to all datasets and some, such as neuronal signatures in the glioma dataset or tissue signatures in teratomas, were specific to individual tumours. Across all tumour types there were closely related clusters of genes associated with cell cycle progression, similar to cell cycle signatures observed previously in other datasets [30,31]. The profile of these genes reflected the known relative proliferative rate of the tumour with, for example, expression at a higher level in aggressive types of ovarian tumours compared to those of a low malignant potential. A prominent cluster found in all graphs was formed of genes associated with the extracellular matrix (ECM); an expression signature also evident in mouse data and analysed with respect to human connective tissue-related diseases [32]. Expression of genes in this cluster was higher in tumours characterised by a fibrotic stroma, for example expression of these genes being elevated in primary mediastinal lymphoma (PMBL) as compared to other subtypes of DLBCL. Other small clusters contained known functional markers of endothelial cells (*PECAM1, EMCN, ESAM, VWF*), smooth muscle cells (*CNN1, MYH11, TPM2*) and adipocytes (*AQP7, CD36, FABP4, LPL*). However, the predominant stromal expression signatures are clearly associated with specific cells or activities of the immune system. Clusters enriched for markers of the monocyte/macrophage (*CD14, CD163, CSF1R*), T cell (*CD2, CD3, CD7, GZMA*), and interferon response (*IFI1-3, MX1/2, OAS1-3*) were present in all tumour types studied here, and their composition was essentially tumour-type independent. There was no evidence of skewing of the macrophage profile towards any particular phenotype. B cell-specific markers and related genes were present in the immune-related networks of some cancers but not others. In all tumours there was a prominent signature representing a post-germinal centre B cell/plasma cell which was rich in immunoglobulin genes as well as markers of post-germinal centre B cells such as *IRF4.* A neutrophil signature was identified only in the colorectal cancer dataset, reflecting the presence histologically of neutrophils in this tumour type.

In all of the graphs there were also many clusters of genes showing relatively uniform expression across samples. Many of the genes that formed these clusters were poorly annotated limiting the possibility of assigning any functional linkage between them but are likely to play a role in uncharacterised cellular housekeeping functions. In mouse data also, cell lineage-specific or inducible expression of genes are associated with more informative annotation, reflecting the priorities of studies on gene function [23].

### Disease networks

Recognised markers associated with specific cancer subtypes often did not fall within large clusters, presumably because they are not highly correlated with global biological features of cancer cells or the associated stroma. For example in the breast cancer dataset, there was a small component of the graph representing genes whose expression is lower in basal-like and ERBB2-positive tumours (Additional file 2). This component included *ESR1* (oestrogen receptor alpha), *GATA3*, *FOXA1* and *XBP1*, and therefore appears to capture a significant proportion of the oestrogen signalling transcriptional network, including modulators and downstream targets. GATA3 is involved in luminal differentiation in normal breast tissue [33] and ESR1 and GATA3 have been demonstrated to reciprocally regulate each other [34]. GATA3 is an inducer of FOXA1, which in turn can induce XBP1, while ESR1 acts both upstream and alongside FOXA1 (reviewed in [35]). Similarly, in the DLBCL dataset, IRF4, one of the markers of the ABC-subtype [6] lies in a sparse network on the edge of the graph, whose nearest neighbours include *FOXP1*, *PIM2* and *CARD11*, all described to be up-regulated in ABC-subtype of DLBCL, with amplifications or mutation affecting *FOXP1*[36] and *CARD11*[36] identified in 38% and 10% respectively of tumours studied (Additional file 3). In both cases it would appear that the graphs have accurately identified key disease modules associated with ESR1 or IRF4 and other genes lying in the immediate neighbourhood merit further investigation. An additional disease module [37], was associated with *SILV* in the skin cancer data [38]. The immediate neighbours of *SILV*, a gene whose product pMel17 is used clinically in the diagnosis of melanoma [39], includes *TYR* (tyrosinase) the key enzyme in melanin biosynthesis, *MLPH* (melanophilin), which plays a role in melanosome transport, *GPR143* expressed on the melanosome membrane and *MLANA* (melan-a). Also present was *MITF*, a melanocytic transcription factor and transcriptional regulator of many of these genes (reviewed in [40]) and *SNCA* (alpha-synuclein).

### Networks constructed based on the mean Pearson correlation values across multiple datasets identify conserved transcriptional signatures

**Figure 5:**
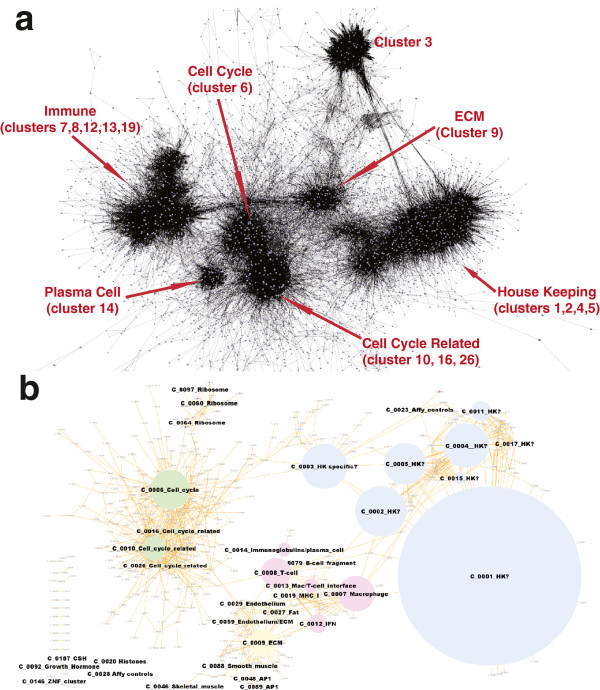
**Network graph of conserved transcription signatures in cancer. a)** 3D graph layout with labelling of main features in the network’s topology. A graph of 9,882 nodes and 184,563 edges was created at Pearson threshold r≥0.6. Clustering of the graph using the MCL algorithm resulted in 639 clusters ranging in size from 1,008 nodes to 4 nodes. A number of large clusters were shown to be highly enriched in genes associated with expression in individual cell types and/or associated with specific cellular functions. See Additional files 3 and 4 for details **b)** Collapsed cluster diagram showing the conserved gene network as a simplified 2D-network where single nodes represent a gene cluster and are sized according to the number of transcripts within the cluster, edges represent connections between members of each cluster. Nodes representing the main clusters have been coloured according to functional groupings. Blue - clusters represent those associated with housekeeping functions; green - clusters of genes which are directly involved in cell cycle progression or whose expression is in way some linked to it; pink - genes associated with the immune component of the tumour and yellow - other stromal elements. Smaller clusters enriched with genes of known function are also shown.

Having examined gene expression signatures within individual datasets, we questioned whether these signatures were preserved across different tumour types. Using six datasets (breast, colonic, DLBCL, glioma, ovarian, testicular) a full correlation matrix was calculated for each and the mean Pearson correlation coefficient across the datasets calculated between all combinations of probes. Layout of a graph derived from the mean Pearson values (*r* = ≥0.6) across the six different tumour types resulted in a smaller graph than those derived from individual tumours at this threshold (Figure 5).

The topology of this graph broke down into three main components which draw together clusters enriched in genes associated with broad functional groupings. The dominant topological feature contained four of the five largest gene clusters. Many of the genes in these clusters are poorly characterised but relatively uniformly expressed across all samples in all datasets and were therefore designated the ‘house-keeping’ (HK) clusters. In general these clusters were poorly conserved in individual datasets although areas of the graphs enriched in housekeeping clusters were clearly identifiable.

A second portion of the graph was highly enriched with genes encoding cell-cycle or cell-cycle related proteins. Cluster 6 contains multiple cyclins, kinesins, members of the minichromosome-maintenance-complex, E2F transcription factor family members, DNA polymerases and topoisomerase. The Aurora kinases (*AURKA, AURKB*), *BUB1* and both checkpoint proteins (*CHEK1* and *2*) are also present along with many other genes that have previously been associated with the cell cycle (see Additional file 4 for the full list of genes/clusters and Additional file 5 for an enrichment analysis of these clusters). Associated with the cell cycle cluster are further smaller clusters e.g. clusters 10, 16 and 26 enriched in mitochondrial, ribosomal and glycolysis-related genes.

The third main area of the graph is clearly associated with different elements of the tumour stroma with a number of immune-related gene clusters in close proximity to each other and those representing other stromal components being somewhat more distant. The macrophage cluster (cluster 7) from the combined cancer graph contains many genes considered to be specific to the myeloid lineage including *CD68, CD14* and *CSF1R*. There is also enrichment for lysosomal genes, multiple genes involved in chemotaxis, and multiple toll-like receptors as well as scavenger receptors *CD163, MARCO, MSR1* which have previously been described by many groups as expressed in tumour-associated macrophages (see [41] for a review). Also, within the macrophage cluster are multiple components of the MHC class II antigen processing machinery. Interestingly, among the genes also expressed is *CD86*, the co-stimulatory molecule, suggesting that these cells may be able to efficiently present antigen to T cells. The T cell cluster (cluster 8) contains pan-T cell markers (*CD2, CD3, CD7*) and elements of the T cell receptor signalling cascade (*ZAP70, LCK, VAV, ITK*). There are many chemokines, cytokines and their receptors in the cluster (*CXCL9, CCL19, CCL5, LTB, CXCR3, CXCR6, CCR7, CCR2, CCR5, IL2RB, IL2RG, IL17R, IL10RA*) including also interferon gamma (IFNG), the prototypical ‘classical’ macrophage activator. The T cell signature is suggestive of an active state with expression of cytotoxic molecules granzymes and perforin as well as markers of activation (*CD69)*. Lying adjacent to the T cell and macrophage clusters is a cluster of genes many of which have been associated with an interferon response containing elements of the proteasome and multiple interferon regulatory factors and interferon inducible proteins.

The largest non-immune-related element of the stromal signature is a cluster of genes associated with extracellular matrix which are almost certainly expressed specifically in fibroblasts/myofibroblasts. It contains structural proteins including many collagens as well as cadherins, laminins, fibrillin and integrins. The signature also contains modifiers of the extracellular matrix such as *MMP2, LOXL1, ADAMTS12, ADAMTS2* and receptors for growth factors (*PDGFRB*) and shares a high degree of overlap with the ECM signature derived from mouse [23,32]. The vascular signature fragments into four small but closely aligned clusters, three of which appear to represent endothelium and the fourth associated with the basement membrane/extracellular matrix component. These clusters contain classical and well characterised markers of vascular differentiation such as *PECAM1* (CD31), *CD34, VWF, KDR* and *CDH5.* In addition, they contain many genes that have been identified as endothelial specific genes by alternative bioinformatic analysis approaches (*ECSCR, EMCN, ROBO4, TEK, EPAS1, GPR116*) [42-45], components of the Notch signalling pathway and other endothelial genes which have been demonstrated in normal and tumour associated endothelium such as *PLVAP*[46]. Finally there is a small cluster that contains many adipocyte specific genes including *ADH1B, ADIPOQ, FABP4* and *LPL*. Other small clusters or groupings of small clusters of note contain the Affymetrix control probes, histone complexes, AP1 transcription factors/early response genes (*JUN, JUNB, FOS, EGR1, EGR3, IER2, NR4A1, NR4A2, ATF3, CTGF* and *DUSP1)* and as mentioned previously somatotrophins (*GH1, CSH1, CSH2, CSHL2*).

### Core signatures are conserved in an unrelated dataset

**Figure 6:**
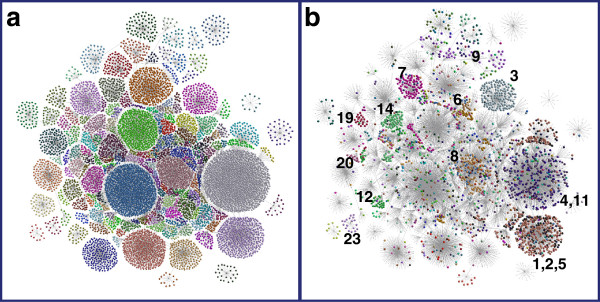
**Conservation of transcriptional signatures in graph derived from skin cancer dataset (Pearson correlation threshold r = 0.80).** In order to provide a clear view of transcripts/clusters within skin cancer graph it has been simplified. The network shown here has been constructed with a central framework of edges derived from the relationships between clusters and nodes representing the transcripts in each cluster joined to a central node representing the cluster with the graph laid out in 2D. Only clusters comprising more than 8 probesets were included. **a)** Colours represent different clusters in the skin cancer data, and **b)** overlay of clusters from the merged cancer (r = 0.6) graph displayed using larger nodes. Many of the housekeeping clusters (1–5, 11) can be seen to be conserved, as is a proportion of the cell cycle (6), macrophage (7), T-cell (8), ECM (9), interferon response (12), plasma cell (14), MHC class 1 (19), histones (20) and Affymetrix control (23) clusters. However it can also be seen that many of the skin cancer clusters are not represented in the merged cancer profile set, these transcriptional signatures being unique to skin cancers.

In order to confirm that the ‘core’ transcriptional signatures generated from the meta-analysis of six datasets are conserved in other cancer datasets, we mapped the signatures onto a number of completely independent tumour datasets derived from skin/melanoma [38], gastric cancer [47] and Hodgkin lymphoma [48]. In each case clusters derived from the meta-analysis of the six tumours identified corresponding clusters in these independent datasets. Shown here are the results of their comparison to a dataset consisting of primary skin cancers including basal cell carcinomas (BCC), squamous carcinomas (SCC) and melanomas, plus a number of metastatic melanomas [38]. Like the other independent datasets, this contained unique transcriptional signatures corresponding to the different tumour types represented in this dataset (Figure 6). However the core signatures were clearly also present. For example, cluster 16 (designated ‘macrophage’) in the skin cancer dataset was highly significantly enriched for genes found in the macrophage cluster in the ‘merged’ dataset (cluster 7 in Figure 5) (adjusted p-value = 1.3^E-120^) implying that these genes represent a true ‘functional unit’, in this case a cell signature. Similarly cell cycle, stromal and house-keeping clusters were also conserved in the skin cancer data (Table 1) and all other cancer datasets so far examined have all generated networks where the conserved signatures identified here have been found to be present.

**Table 1:** Summary of the gene coexpression clusters conserved across all datasets studied here

***Descriptive class***	***Cluster description***	***Cluster ID number***	***No. of probesets in cluster***	***No. of genes in cluster***	***Known markers present in cluster***	***Gene ontology annotation***	***Other annotation***	***Conservation of signature in skin dataset: Cluster number***	***Conservation of signature in skin dataset: significance of enrichment***
							* KEGG pathway, ** Curated gene set, *** Swiss-Prot		
						***(p-value for EASE score)***	**Keywords *(p-value)***		
									***(Adjusted Fisher’s test)***
Immune	Macrophage	7	220	163	CD68, CD14, CD163, CSF1R, Fc Receptors (CD16, CD32, CD64), MHC II molecules	Immune system process (3.96^e-32)^		16	1.3^E-120^
						Defence response (8.79^e-22^)			
	T cell	8	181	145	CD2, CD3, CD6, CD7, CD52, TCR	Immune system process (4.99^e-35^)	TCR signalling pathway (6.1^e-25^) *	6	3.29^E-93^
						Signal transduction (7.32^e-18^)			
						T cell activation (2.58^e-19^)			
	Macrophage/T cell interface	13	87	58		Immune response (7.20^e-06^)		34	2.6^E-13^
	IFN response	12	115	73	GBP1. IFI27, IFR1, IRF2, OAS1, SP100, STAT1,	Immune response (4.32^e-26^)	Genes upregulated by IFNB in HT1080 (1.48^e-47^)**	51	4.06^E-56^
						Response to virus (1.15^e-21^)			
							Genes upregulated by IFNA in HT1080 (1.05^e-43^)**		
	MHC class I	19	35	16	HLA-A, HLA-B, HLA-C, HLA-E, B2M	Antigen processing and presentation (5.51^e-17^)	MHC 1 (9.29^e-15^)***	82	1.2^E-58^
	Ig/ plasma cell	14	85	36	Ig light and heavy chains,	Immune response (7.49^e-10^)	Ig C region (1.12^e-10^)	21	2.48^E-129^
	B-cell	79, 142	10, 7	6, 6	CD19, CD20, CD79	B-cell receptor complex (2.31^e-06^)	BCR signalling (1.28^e-09^)*	6	6.52^E-17^
						Immune response (1.84^e-08^)			
	Mast cell	93	9	4	Tryptase, Fc receptor for IgE	Proteolysis (0.008)	Zymogen (4.77^e-04^)***	167	2.72^E-27^
	AP1 response	48, 89	13, 9	11, 6	FOS, JUNB,	Sequence specific DNA binding (2.19^e-06^)	DNA binding (2.75^e-09^)***	-	-
						Regulation of cellular process (1.2^e-04^)			
Stroma	Extracellular matrix	9	163	100	BGN, CALD1, FN1, collagens	Extracellular matrix (1.93^e-40^)		88	5.5^E-34^
						Cell adhesion (1.16^e-17^)			
	Adipocyte	27	22	15	ADIPOQ, LPL	Response to wounding (0.004)	PPAR signalling (1.46^e-07^)*	-	-
							Adipocyte vs Fibroblast upregulated (1.49^e-15^)**		
	Endothelium	29,38,49	20, 17, 13	17, 13, 11	CD31, CD34, Endomucin, Endoglin, vWF	Cell adhesion (1.3^e-08^)	Upregulated in glomerul in DM vs normal (8.33^e-14^)**	58	6.75^E-16^
						Blood vessel development (1.28^e-10^)			
							Brentani_Angiogenesis (6.87^e-08^)**		
	Endothelium/ECM	59	11	6	COL4A1, COL4A2	Cell adhesion (4.13^e-05^)		215	1.22^E-12^
	Smooth muscle	88, 249	9,6	5, 4	Alpha SMA, calponin	Smooth muscle contraction (4.75^e-05^)	Muscle protein (3.95^e-08^)***	907	1.27^E-11^
	Skeletal muscle	46	15	15	Myoglobin, CKm, Myosin	Contractile fibre part (1.53^e-17^)		23	2.55^E-31^
						Muscle development (2.09^e-05^)			
Cell cycle	Cell cycle	6	239	182	AURKA, BUB1, CHEK2, CDC2, MCM2	Cell cycle (3.06^e-59^)	Serum fibroblast cell cycle (1.17e-117)**	101	1.31^E-26^
						DNA replication (1.48^e-39^)			
	Cell cycle related	10, 16, 26	147, 52, 23	125, 44, 19		RNA binding (2.29^e-11^)	RNA binding (2.29^e-14^)***	-	-
Ribosomal	Ribosomal	54, 60, 64, 97	12, 11, 11, 8	12, 8, 6, 5	RPL38, RPS10, RPS19	Cytosolic ribosome (7.91^e-13^)	Ribosomal protein (8.44^e-11^)***	54	4.25^E-36^
							Protein biosynthesis (1.62^e-06^)***		
Other functional classes	Histones	20	30	26	HIST1H1C, HIST1H2AB, HIST1H3H	Nucleosome (9.75^e-23^)	Nucleosome core (2.61^e-21^)***	160	9.15^E-35^
						Chromatin assembly (9.24^e-21^)			
	Glycolysis	47	13	5	GAPDH, GPI	Glycolysis (1.46^e-05^)	Gluconeogenesis (9.37^e-07^)***	151	6^E-30^
	Haemoglobins	91	9	2	HBA1, HBB			144	6^E-31^
Affymetrix controls	Affymetrix controls	23, 28	26, 22	26, 22				99	4.57^E-59^

Each cluster or group of related clusters has been placed into a functional grouping based on the biology from which it is derived. Details of the cluster(s) are provided together with selected pathway/Gene ontology enrichment scores for the genes that make up the clusters. (For a complete list see Additional file 4).

This study demonstrates the feasibility of an *in silico* alternative to laser capture microscopy to identify the gene expression profiles of the cells that make up a tumour. Understanding the microenvironment of the tumour allows exploration of potential new targets for therapy, directed not at the malignant cells, but at the environment in which they exist. Study of these cells is complicated by the heterogeneous background in which they exist and isolation of the cells from their background, unless by approaches such as microdissection, will inevitably change them. Any clinically relevant approach to the microenvironment must of necessity, address the microenvironment of the established tumour, rather than the factors that contribute to tumour development. We have used BioLayout *Express*^3D^ a visualization tool that allows exploration of complex networks of a size not previously possible [21]. Furthermore, we have used the MCL algorithm [27] to group nodes (genes) into clusters in a completely unsupervised manner. In this respect MCL has been shown to perform as well or better than other network clustering algorithms [49]. Previous efforts to identify gene signatures/modules in cancer data have used different analytical approaches, less data, grouped far fewer genes and often failed to explain the biological significance of their findings [9-11,50,51]. Where correlation networks have been used previously to analyse modularity in gene expression data [13,52], available computing frameworks have not permitted the visualization or exploration of the resultant graphs as effectively in the current study. The robustness across different datasets, and the obvious association of genes of known function or cell lineage-restriction, provides a strong internal validation for our approach.

The preservation of specific clusters associated with stromal cells across such a large number of genetically diverse individuals and multiple tumour types argues there is a common tumour microenvironment that controls, and is controlled by, interactions amongst elements of the stroma. There is already a wealth of data on the role of the tumour associated macrophage (TAM), with the majority of studies suggesting that large numbers of TAMs are associated with poor prognosis (reviewed in [53,54]). Macrophages have been attributed functions in assisting invasion, promoting angiogenesis and subverting an immune response to the advantage of the tumour. To date there are only global gene expression profiles from TAMs derived from inbred mouse tumour models in which the cells have been separated from their microenvironment and therefore potentially had their gene expression altered by the process of isolation. Our core macrophage signature contains genes involved in phagocytosis, MHC class II antigen presentation and T cell co-stimulation and is therefore suggestive of a macrophage acting as an antigen-presenting cell. The TAM profile also contains scavenger receptors and genes involved in lipid metabolism suggesting a role in apoptotic cell clearance by TAMs. Analysis of the T cell profile demonstrates the presence of an almost completely intact antigen recognition and signalling pathway containing elements of the TCR, co-receptors and downstream signalling molecules. Also in the signature are cytotoxic molecules and markers of activation suggesting these are, at least in part, activated cytotoxic T cells. The preservation across all tumour types of a cluster of genes associated with an interferon response and the presence of *IFNG* in the T cell signature argues that activation of this pathway forms a consistent part of the response to a tumour. This is also in keeping with data derived from murine models in which it was shown that TAMs express many interferon inducible genes [55]. Taken together, these data do not support the view that TAMs have a so-called M2 (or alternative activation) [19] phenotype characterised by dominant actions of interleukin 4. Nor does the analysis support the view that recognition of tumour-associated antigens is compromised by a lack of antigen-presenting cells.

One of the larger signatures observed is associated with the extracellular matrix. This was enriched in structural proteins, proteoglycans, modifiers of the extracellular matrix and signalling molecules. Histologically, the presence of a desmoplastic tumour stroma is a well recognised phenomenon occurring in many tumour types. However, like many other elements of the microenvironment the precise role played by this reactive stroma has been difficult to assess: is the role of the stroma to contain the tumour or is it yet another factor recruited to promote the survival of the malignant cells? Recent data from studies of DLBCL suggest that in this tumour at least the answer may be that different elements of the stroma contribute to both a good and a poor prognosis [56], whereas work in small cell carcinoma has established the role of interactions between the ECM and tumour cells in resisting chemotherapy-induced death [57]. More recently work in a lung carcinoma model [58] highlighted the role that ECM components, in this case versican, can play in activating other elements of the microenvironment suggesting that as for other elements of the tumour microenvironment, cross-talk between elements is likely to be of great importance.

The vasculature signature observed here contains many well characterised markers of endothelial cells as well as less well characterised endothelial genes. It contains receptors and co-receptors (*KDR, NRP2*) for VEGF, the major angiogenic factor but also contains elements associated with Notch signalling, another important system in angiogenesis (for a review see [59]). *NOTCH3*, usually expressed in vascular smooth muscle, lies in the endothelial-related cluster enriched in ECM and basement membrane proteins. A recent study investigated the crosstalk between endothelial and mural cells via NOTCH3 signalling and showed a reduction in angiogenesis in an *in vitro* co-culture system when *NOTCH3* is knocked out in mural cells [60].

The fact that macrophage, T cell, ECM and endothelial-specific genes form independent clusters, indicates that there is not a tight causal relationship between them. The T cell signature and macrophage signatures are to some extent correlated in all of the networks, but this does not necessarily imply an interaction beyond the fact that the most fibrotic regions of a tumour tend to exclude leukocytes so the two cell types could be co-enriched by chance. Despite the reported association of macrophage number with microvessel density in some solid tumours [61-64], the signatures of macrophages and endothelial cells are clearly separate, so there is not likely to a strict macrophage requirement for angiogenesis, and in fact the drive to angiogenesis is likely to be multifactorial. This viewpoint is supported by the fact that the macrophage cluster does not contain any of the known regulators of endothelial proliferation, such as the vascular endothelial growth factors (VEGFs). Neither macrophage nor endothelial cluster contains *TIE2*, which has been implicated, based largely upon *in vitro* studies, in tumour-associated angiogenesis and regulatory T cell production [65,66]. Indeed, the T cell cluster does not contain *FOXP3* or *CD25*, indicating that regulatory T cell activation is not a ubiquitous feature of the immune environment of tumours.

## Conclusion

In summary, we have demonstrated a unique approach to phenotyping cell types and identifying pathways within cancer without the need for technologies such as microdissection. The approach is related to the views of pathways, interaction and functional relationship that can be derived from analysis of introduced genetic variation in yeast [67]. The core signatures we report provide a tool to aid the analysis of further datasets, and using the tool BioLayout *Express*^3D^ they can readily be overlaid on to other data as an aid to the interpretation of other large-scale expression data. In considering therapeutic approaches to cancer, our approach identifies sets of genes that are common to a range of tumour types, and to the stromal components, and which might therefore be potential targets. It also identifies candidate markers for assessing the mechanism and efficacy of therapeutic intervention.

## Methods

### Selection and preprocessing of datasets

Datasets were selected on the following criteria; analysis of primary human tumour samples, large study size, availability of raw data with provision of clinical annotation and genome-wide analysis using the Affymetrix U133 platforms (either U133A + B or U133Plus2.0). These datasets were identified from Gene Expression Omnibus (GEO) or caArray and CEL files downloaded. See Table 2 for details.

**Table 2:** List of the cancer datasets used for this study

**Database reference**	**Reference (PMID)**	**Tumor type(s)**	**Cases analysed**	**Affymetrix U133 Platform(s)**	**Graph size (nodes)**	**Graph size (edges)**
GSE11318	Lenz et al. (18765795)	DLBCL	194	Plus 2.0	19,850	614,273
GSE1456	Pawitan et al. (16280042)	Breast carcinoma	134	A & B	19,246	559,761
GSE9891	Tothill et al. (18698038)	Ovarian (epithelial) carcinoma	265	Plus 2.0	19,415	268,471
GSE3218	Korkola et al. (16424014)	Testicular germ cell tumours	86	A & B	18,934	954,082
GSE13294	Jorissen et al. (19088021)	Colorectal carcinoma	150	Plus 2.0	22,687	725,467
caArray/rembr-00037	REMBRANT – Repository for Molecular Brain Neoplasia Data	Primary CNS tumours	253	Plus 2.0	23,015	623,591
**GSE7553**	**Riker et al. (18442402)**	**Skin tumours**	**77**	**A** &**B**	**19,623**	**600,143**
**GSE17920**	**Steidl et al. (20220182)**	**Hodgkin lymphoma**	**131**	**Plus 2.0**	**13,846**	**521,593**
**GSE15459**	**Ooi et al. (19798449)**	**Gastric cancer**	**200**	**Plus 2.0**	**15,747**	**719,884**

The datasets with a white background are the six used for the primary analysis and the remaining three (bold) the datasets used to confirm the robustness of the core cancer expression signatures.

The initial analysis used six individual datasets. The breast cancer dataset [4] was stratified on the basis of molecular tumour type (basal, HER2 positive, luminal A or B, or normal-like), Ellis-Elston grade, and outcome. The colorectal dataset [68] was divided into microsatellite stable and unstable tumours. The lymphoma dataset [69] was stratified into germinal centre B cell-like (GCB), activated B cell-like (ABC), primary mediastinal B cell (PMBL) and unclassified, based on gene expression and further organised on the basis of sex and patient outcome. The glioma dataset, derived from caArray, was stratified on the basis of histological type (astrocytoma, oligodendroglioma, glioblastoma), WHO grade and sex. The ovarian dataset [70] was stratified by malignant or low malignant potential, histological type (endometrioid or serous), grade, stage and primary site (ovary, peritoneum or fallopian tube). The testicular dataset [71] contained primary germ cell tumours and was stratified by pure or mixed histological types and then within each category on the constituent elements using the WHO classification (seminoma, teratoma, embyronal carcinoma, yolk sac tumour, choriocarcinoma). As such these data were selected to represent a broad range of tumour biology. Other data derived from various types of skin cancer including BCC, SCC, primary melanoma, and melanoma metastatic to subcutaneous tissue, lymph node, brain and adrenal gland [38], gastric cancer [47] and Hodgkins lymphoma [48] were used as test datasets to verify the conservation of gene signatures in independent datasets.

**Figure 7:**
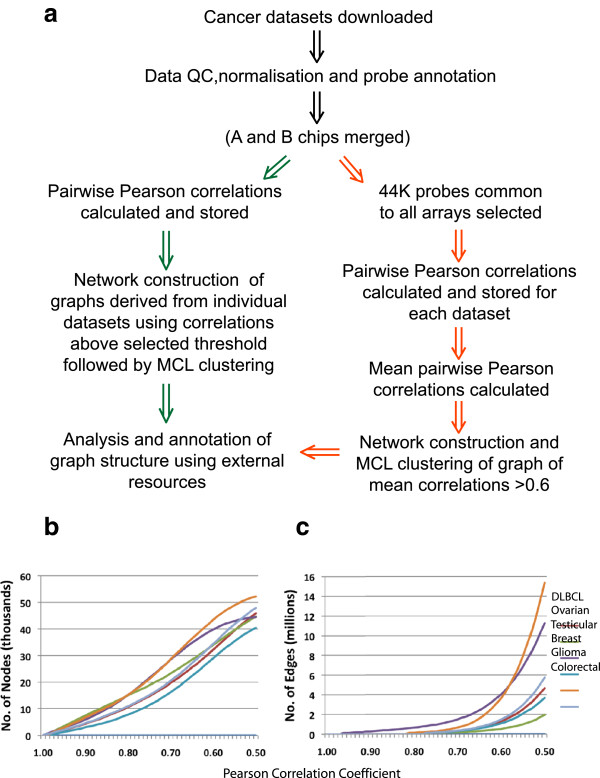
**Approach to data analysis. a)** Flow diagram summarising the analysis pipeline used here. The size of graph generated from each dataset at different Pearson correlation thresholds in terms of **b)** the number of nodes and **c)** number of edges.

A summary of the data analysis pipeline is shown in Figure 7a. The quality of the raw data from each dataset was reanalysed using the arrayQualityMetrics package in Bioconductor (http://www.bioconductor.org/) and scored on the basis of 5 metrics, namely maplot, spatial, boxplot, heatmap and rle [72]. Any array failing on more than one metric was removed (Table 2) and in cases comprising A and B arrays, failure of one chip resulted in removal of data for that patient from analysis. Where the data was derived from A and B arrays, a single merged file was created for each sample. Following QC, each dataset was normalised independently using the robust multi-array average (RMA) expression measure [73]. Probesets were annotated using Bioconductor (26 June 2009) and samples ordered according to clinical grouping.

### Network analysis

Each dataset was saved as an ‘.expression’ file containing a unique identifier for each row of data (Gene symbol concatenated to probeset ID), followed by columns of gene annotations used as class-sets for the overlay and analysis of information with respect to the graph, and finally natural scale normalised data values for each sample (each column of data being derived from a different sample). These files were then loaded into the network analysis tool BioLayout *Express*^3D^[21]. Pairwise Pearson correlations were calculated for each probeset on the array(s) as a measure of similarity between the signal derived from different probesets. All Pearson correlations where *r* ≥ 0.6 were saved to a ‘.pearson’ file. Graph layout was performed using a modified Fruchterman-Rheingold algorithm [74] in 3-dimensional space in which nodes representing genes/transcripts are connected by weighted, undirected edges representing correlations above the selected threshold. Depending on the size of the dataset and the inherent variation of samples, datasets produced graphs of varying sizes at a given correlation threshold value (Figure 7b). Selected correlation thresholds for individual datasets were designed to include approximately 40% of the available data (Table 2). The resultant graphs were very large and highly structured (Figure 3). The topology of the graph contained localised areas of high connectivity and high correlation (representing clusters of genes with similar profiles), were determined using the Markov Cluster (MCL) algorithm which simulates multiple iterations of random flow through the graph structure. An MCL inflation value of 2.2 was used as the basis of determining the granularity of clustering, as it has been shown to be optimal when working with highly structured expression graphs [21]. Clusters were named according to their relative size, the largest cluster being named cluster 1. Graphs of each dataset were explored extensively in order to understand the significance of the gene clusters and their relevance to the pathology of the tumours.

### Cluster annotation

Gene set enrichment analysis was performed on clusters using DAVID [30,75] and GSEA MSigDB [31] web-based analysis tools to determine the significance of co-expressed genes. Clusters were annotated if hits of high significance showed a common trend as to function. These analyses were supplemented by comparison of the clusters with tissue- and cell-specific clusters derived from network-based analyses of a human tissue atlas and an atlas of purified leukocyte populations [28,29] and comprehensive reviews of the literature.

### Comparison of expression patterns across Six cancers and validation of signatures

To allow direct comparison of the datasets, probesets that were not represented in all datasets i.e. were only present on the U133Plus 2.0 array, were removed leaving 44,754 probesets common to all U133 platforms. The Pearson correlation coefficient between data derived from each probe-set in individual datasets was calculated using BioLayout *Express*^3D^ and all correlation values written to file. A mean Pearson correlation was then calculated for each transcript i.e. the average correlation between probesets across the six datasets. Mean Pearson correlations were filtered to remove any values below a user defined threshold and network graphs constructed. The work described here is based on the analysis of a graph constructed using a mean Pearson threshold of *r* ≥0.6 and clustered with an MCL inflation value of 2.2.

In order to analyse the conservation of the gene signatures in an unrelated dataset, the clusters and associated annotations derived from the merged *r* ≥0.6 graph were mapped onto to datasets derived from various types of skin cancer [38], gastric cancer [47] and Hodgkin’s lymphoma [48]. Graphs were constructed from these data and clustered, and these clusters were analysed for enrichment of genes from the ‘core signatures’ using Fisher’s test with an adjustment for multiple of testing.

## Competing interests

The authors declare that they have no competing interests.

## Authors’ contributions

TND performed the network analysis of data, was the primary author of the work and brought a Clinical Histopathologist’s perspective to the interpretation of the data. DAH helped conceive the work and was a primary author of the paper. TT was the primary developer of the network analysis tool and where required implemented a number of new features in support of the work. JRG and CDG oversaw the work, provided input into the interpretation of the gene signatures and contributed to the writing of the paper. TCF helped conceive the idea, was instrumental in organizing the collection of the data, guided the network analysis of the data and was a primary author of the paper. All authors read and approved the final manuscript.

## Supplementary Material

Additional file 1 Table of macrophage gene cluster derived from DLBCL dataset when examined using a Pearson correlation cut off of ≥0.65 and clustered using an MCL inflation value of 2.2. Click here for file

Additional file 2 **Coexpression clustering of genes associated with *ESR1 *in breast cancer dataset.** As expected the expression of *ESR1* (below) shows a marked reduction in ER-negative tumours. Examination of the neighbours of *ESR1* in the correlation network pulls out many of the known *ESR1* targets including FOXA1, XBP1, ERBB4 and GATA3 together with some new and interesting candidate genes. Click here for file

Additional file 3 **Coexpression clustering of genes associated with *IRF4 *in DLBCL dataset.** IRF4, one of the markers of the ABC-subtype [6] lies in a sparse network on the edge of the graph. Its nearest neighbours include FOXP1, PIM2 and CARD11, all described to be up-regulated in ABC-subtype of DLBCL, with amplifications or mutation affecting FOXP1 and CARD11 identified in 38% and 10% respectively of tumours studied. Click here for file

Additional file 4 **Cluster analysis of merged cancer (r = 0.6) graph.** This table lists the gene membership and annotation of each cluster from the meta-analysis of gene expression data derived from multiple tumour types. Click here for file

Additional file 5 **GO analysis of the main clusters of interest from the combined cancer data analysis.** All graphs and tables described in this work are available from the website http://www.OncoGraph.org which supports the direct visualization of graphs in BioLayout *Express*^3D^ using Java web start technology. Click here for file
