# Supplementary material for: Coexpression analysis of large cancer datasets provides insight into the cellular phenotypes of the tumour microenvironment
Source: BMC Genomics. 2013 Jul 11;14:469. doi: 10.1186/1471-2164-14-469 (PMC3721986; doi:10.1186/1471-2164-14-469)
Supplement: Additional file 1 — Table of macrophage gene cluster derived from DLBCL dataset when examined using a Pearson correlation cut off of ≥0.65 and clustered using an MCL inflation value of 2.2. [file 1471-2164-14-469-S1.docx]

Genes present in the ‘macrophage’ signature of the DLBCL dataset at Pearson ≥0.65 and MCL 2.2.

| ACP2 | acid phosphatase 2, lysosomal |
| --- | --- |
| ADAMDEC1 | ADAM-like, decysin 1 |
| ADAP2 | ArfGAP with dual PH domains 2 |
| ADORA3 | adenosine A3 receptor |
| AIF1 | allograft inflammatory factor 1 |
| AOAH | acyloxyacyl hydrolase (neutrophil) |
| APOL1 | apolipoprotein L, 1 |
| APOL2 | apolipoprotein L, 2 |
| APOL3 | apolipoprotein L, 3 |
| ARRB2 | arrestin, beta 2 |
| ATP8B4 | ATPase, class I, type 8B, member 4 |
| BCL2A1 | BCL2-related protein A1 |
| C1orf38 | chromosome 1 open reading frame 38 |
| C1orf54 | chromosome 1 open reading frame 54 |
| C1QA | complement component 1, q subcomponent, A chain |
| C1QB | complement component 1, q subcomponent, B chain |
| C1QC | complement component 1, q subcomponent, C chain |
| C2 | complement component 2 |
| C3AR1 | complement component 3a receptor 1 |
| C5AR1 | complement component 5a receptor 1 |
| CAPG | capping protein (actin filament), gelsolin-like |
| CCR1 | chemokine (C-C motif) receptor 1 |
| CCRL2 | chemokine (C-C motif) receptor-like 2 |
| CD14 | CD14 molecule |
| CD163 | CD163 molecule |
| CD300A | CD300a molecule |
| CD4 | CD4 molecule |
| CD68 | CD68 molecule |
| CD74 | CD74 molecule, major histocompatibility complex, class II invariant chain |
| CD84 | CD84 molecule |
| CD86 | CD86 molecule |
| CD97 | CD97 molecule |
| CECR1 | cat eye syndrome chromosome region, candidate 1 |
| CHIT1 | chitinase 1 (chitotriosidase) |
| CIITA | class II, major histocompatibility complex, transactivator |
| CLEC10A | C-type lectin domain family 10, member A |
| CLEC2B | C-type lectin domain family 2, member B |
| CLEC7A | C-type lectin domain family 7, member A |
| CMKLR1 | chemokine-like receptor 1 |
| CSF1R | colony stimulating factor 1 receptor |
| CSF2RB | colony stimulating factor 2 receptor, beta, low-affinity (granulocyte-macrophage) |
| CTSB | cathepsin B |
| CTSC | cathepsin C |
| CTSD | cathepsin D |
| CTSL1 | cathepsin L1 |
| CTSS | cathepsin S |
| CYBB | cytochrome b-245, beta polypeptide |
| CYTH4 | cytohesin 4 |
| DPYD | dihydropyrimidine dehydrogenase |
| DRAM | damage-regulated autophagy modulator |
| DSE | dermatan sulfate epimerase |
| EMR2 | egf-like module containing, mucin-like, hormone receptor-like 2 |
| FCER1G | Fc fragment of IgE, high affinity I, receptor for; gamma polypeptide |
| FCGR1A | Fc fragment of IgG, high affinity Ia, receptor (CD64) |
| FCGR1B | Fc fragment of IgG, high affinity Ib, receptor (CD64) |
| FCGR2A | Fc fragment of IgG, low affinity IIa, receptor (CD32) |
| FCGR3B | Fc fragment of IgG, low affinity IIIb, receptor (CD16b) |
| FGL2 | fibrinogen-like 2 |
| FGR | Gardner-Rasheed feline sarcoma viral (v-fgr) oncogene homolog |
| FPR1 | formyl peptide receptor 1 |
| FPR3 | formyl peptide receptor 3 |
| GBP2 | guanylate binding protein 2, interferon-inducible |
| GCH1 | GTP cyclohydrolase 1 |
| GPNMB | glycoprotein (transmembrane) nmb |
| GPR65 | G protein-coupled receptor 65 |
| GPX1 | glutathione peroxidase 1 |
| HCK | hemopoietic cell kinase |
| HK3 | hexokinase 3 (white cell) |
| HLA-DMA | major histocompatibility complex, class II, DM alpha |
| HLA-DMB | major histocompatibility complex, class II, DM beta |
| HLA-DOA | major histocompatibility complex, class II, DO alpha |
| HLA-DPA1 | major histocompatibility complex, class II, DP alpha 1 |
| HLA-DPB1 | major histocompatibility complex, class II, DP beta 1 |
| HLA-DQA1 | major histocompatibility complex, class II, DQ alpha 1 |
| HLA-DQB1 | major histocompatibility complex, class II, DQ beta 1 |
| HLA-DRA | major histocompatibility complex, class II, DR alpha |
| HLA-DRB1 | major histocompatibility complex, class II, DR beta 1 |
| HLA-DRB4 | major histocompatibility complex, class II, DR beta 4 |
| HLA-DRB5 | major histocompatibility complex, class II, DR beta 5 |
| HLA-DRB6 | major histocompatibility complex, class II, DR beta 6 (pseudogene) |
| HS3ST2 | heparan sulfate (glucosamine) 3-O-sulfotransferase 2 |
| IFI30 | interferon, gamma-inducible protein 30 |
| IGSF6 | immunoglobulin superfamily, member 6 |
| IL15RA | interleukin 15 receptor, alpha |
| IL18BP | interleukin 18 binding protein |
| ITGAM | integrin, alpha M (complement component 3 receptor 3 subunit) |
| ITGAX | integrin, alpha X (complement component 3 receptor 4 subunit) |
| ITGB2 | integrin, beta 2 (complement component 3 receptor 3 and 4 subunit) |
| LACTB | lactamase, beta |
| LAIR1 | leukocyte-associated immunoglobulin-like receptor 1 |
| LAPTM5 | lysosomal multispanning membrane protein 5 |
| LCP2 | lymphocyte cytosolic protein 2 (SH2 domain containing leukocyte protein of 76kDa) |
| LGALS9 | lectin, galactoside-binding, soluble, 9 |
| LGMN | legumain |
| LHFPL2 | lipoma HMGIC fusion partner-like 2 |
| LILRB1 | leukocyte immunoglobulin-like receptor, subfamily B (with TM and ITIM domains), member 1 |
| LILRB2 | leukocyte immunoglobulin-like receptor, subfamily B (with TM and ITIM domains), member 2 |
| LILRB4 | leukocyte immunoglobulin-like receptor, subfamily B (with TM and ITIM domains), member 4 |
| LIPA | lipase A, lysosomal acid, cholesterol esterase |
| LST1 | leukocyte specific transcript 1 |
| LY86 | lymphocyte antigen 86 |
| LY96 | lymphocyte antigen 96 |
| LYZ | lysozyme (renal amyloidosis) |
| MAFB | v-maf musculoaponeurotic fibrosarcoma oncogene homolog B (avian) |
| MAN2B1 | mannosidase, alpha, class 2B, member 1 |
| MARCO | macrophage receptor with collagenous structure |
| MFSD1 | major facilitator superfamily domain containing 1 |
| MGAT1 | mannosyl (alpha-1,3-)-glycoprotein beta-1,2-N-acetylglucosaminyltransferase |
| MNDA | myeloid cell nuclear differentiation antigen |
| MPP1 | membrane protein, palmitoylated 1, 55kDa |
| MS4A4A | membrane-spanning 4-domains, subfamily A, member 4 |
| MS4A6A | membrane-spanning 4-domains, subfamily A, member 6A |
| MS4A7 | membrane-spanning 4-domains, subfamily A, member 7 |
| MSR1 | macrophage scavenger receptor 1 |
| MYO1F | myosin IF |
| NAGK | N-acetylglucosamine kinase |
| NCF2 | neutrophil cytosolic factor 2 |
| NCKAP1L | NCK-associated protein 1-like |
| NPL | N-acetylneuraminate pyruvate lyase (dihydrodipicolinate synthase) |
| NR1H3 | nuclear receptor subfamily 1, group H, member 3 |
| P2RY13 | purinergic receptor P2Y, G-protein coupled, 13 |
| PILRA | paired immunoglobin-like type 2 receptor alpha |
| PLA2G7 | phospholipase A2, group VII (platelet-activating factor acetylhydrolase, plasma) |
| PLCB2 | phospholipase C, beta 2 |
| PLEK | pleckstrin |
| PLEKHO2 | pleckstrin homology domain containing, family O member 2 |
| PTAFR | platelet-activating factor receptor |
| RHOG | ras homolog gene family, member G (rho G) |
| RNASE6 | ribonuclease, RNase A family, k6 |
| RNF130 | ring finger protein 130 |
| SCO2 | SCO cytochrome oxidase deficient homolog 2 (yeast) |
| SCPEP1 | serine carboxypeptidase 1 |
| SECTM1 | secreted and transmembrane 1 |
| SIGLEC1 | sialic acid binding Ig-like lectin 1, sialoadhesin |
| SIGLEC7 | sialic acid binding Ig-like lectin 7 |
| SLAMF8 | SLAM family member 8 |
| SLC15A3 | solute carrier family 15, member 3 |
| SLC31A2 | solute carrier family 31 (copper transporters), member 2 |
| SLC7A7 | solute carrier family 7 (cationic amino acid transporter, y+ system), member 7 |
| SLCO2B1 | solute carrier organic anion transporter family, member 2B1 |
| SNX10 | sorting nexin 10 |
| SPI1 | spleen focus forming virus (SFFV) proviral integration oncogene spi1 |
| SRGN | serglycin |
| TBXAS1 | thromboxane A synthase 1 (platelet) |
| TCIRG1 | T-cell, immune regulator 1, ATPase, H+ transporting, lysosomal V0 subunit A3 |
| TFEC | transcription factor EC |
| TLR1 | toll-like receptor 1 |
| TLR2 | toll-like receptor 2 |
| TLR7 | toll-like receptor 7 |
| TLR8 | toll-like receptor 8 |
| TM6SF1 | transmembrane 6 superfamily member 1 |
| TMEM140 | transmembrane protein 140 |
| TNFAIP2 | tumor necrosis factor, alpha-induced protein 2 |
| TNFRSF14 | tumor necrosis factor receptor superfamily, member 14 (herpesvirus entry mediator) |
| TNFRSF1B | tumor necrosis factor receptor superfamily, member 1B |
| TNFSF13B | tumor necrosis factor (ligand) superfamily, member 13b |
| TREM2 | triggering receptor expressed on myeloid cells 2 |
| TRPV2 | transient receptor potential cation channel, subfamily V, member 2 |
| TYMP | thymidine phosphorylase |
| TYROBP | TYRO protein tyrosine kinase binding protein |
| VAMP5 | vesicle-associated membrane protein 5 (myobrevin) |
| VSIG4 | V-set and immunoglobulin domain containing 4 |
