# Supplementary figures and images for: Coexpression analysis of large cancer datasets provides insight into the cellular phenotypes of the tumour microenvironment
Source: BMC Genomics. 2013 Jul 11;14:469. doi: 10.1186/1471-2164-14-469 (PMC3721986; doi:10.1186/1471-2164-14-469)

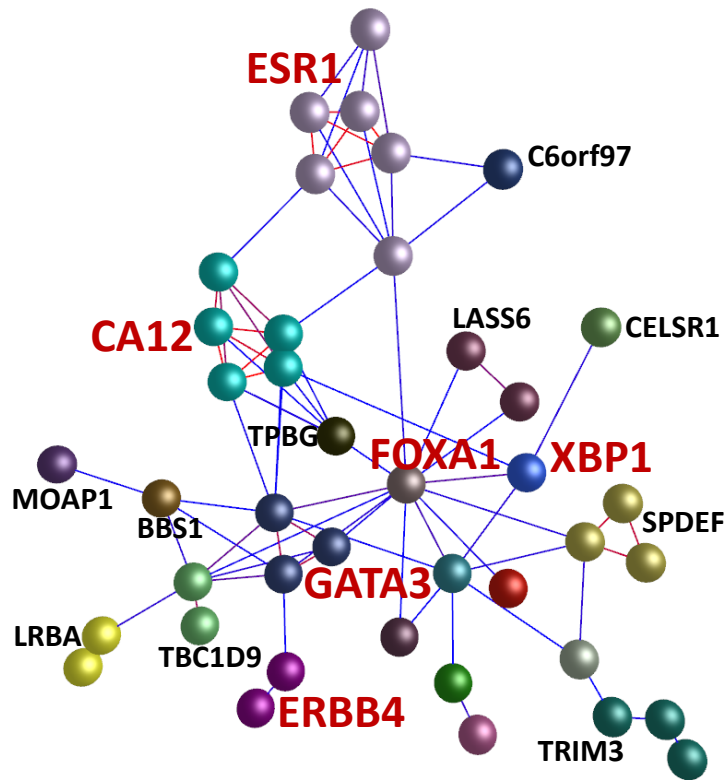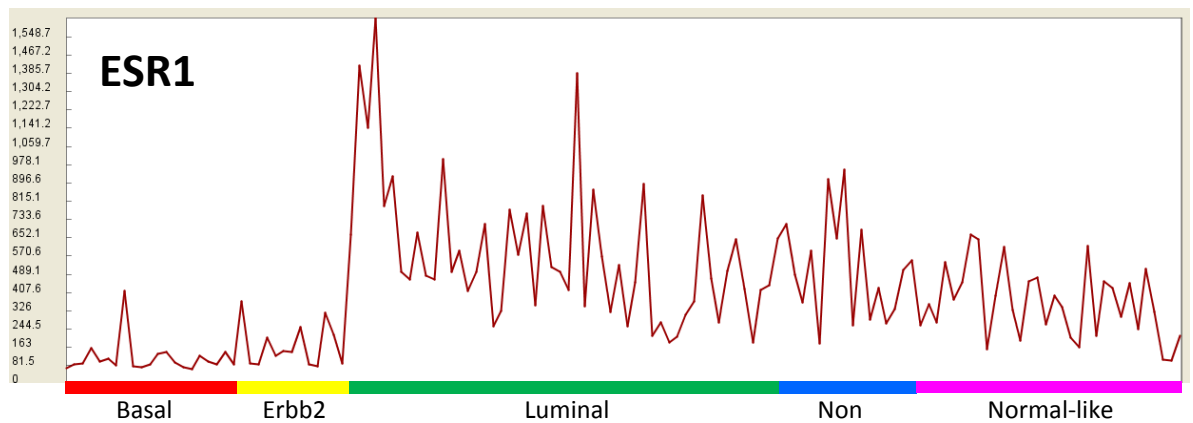

Supplement: Additional file 2 — Coexpression clustering of genes associated with ESR1 in breast cancer dataset. As expected the expression of ESR1 (below) shows a marked reduction in ER-negative tumours. Examination of the neighbours of ESR1 in the correlation network pulls out many of the known ESR1 targets including FOXA1, XBP1, ERBB4 and GATA3 together with some new and interesting candidate genes. [file 1471-2164-14-469-S2.pdf]

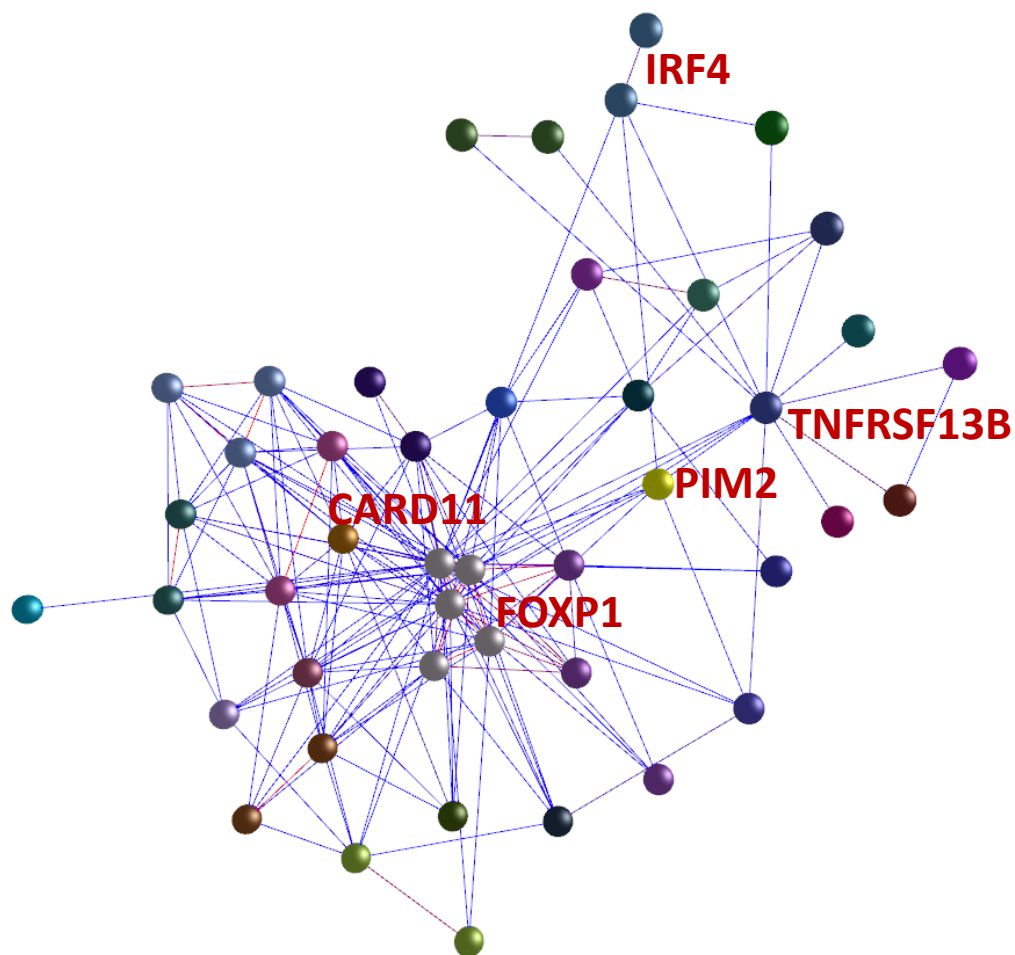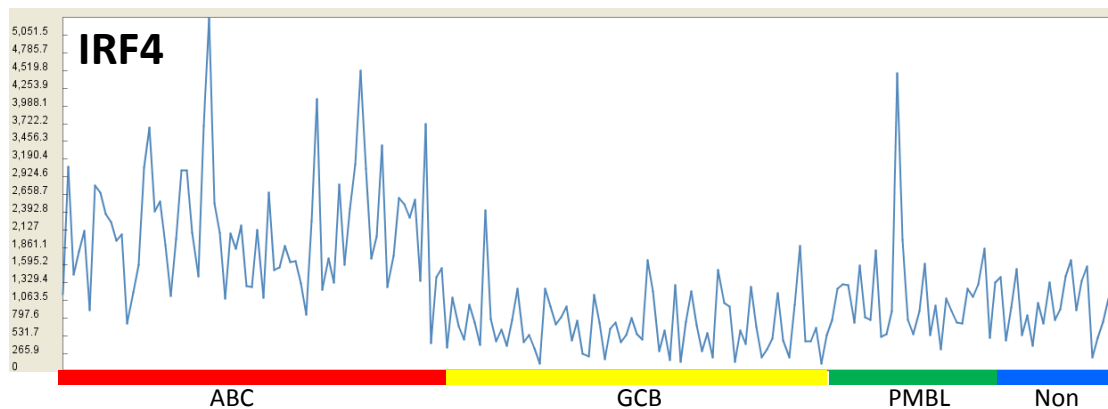

Supplement: Additional file 3 — Coexpression clustering of genes associated with IRF4 in DLBCL dataset. IRF4, one of the markers of the ABC-subtype [6] lies in a sparse network on the edge of the graph. Its nearest neighbours include FOXP1, PIM2 and CARD11, all described to be up-regulated in ABC-subtype of DLBCL, with amplifications or mutation affecting FOXP1 and CARD11 identified in 38% and 10% respectively of tumours studied. [file 1471-2164-14-469-S3.pdf]
